# Essentials of Dermatology

**Published:** 1986-04

**Authors:** A. T. M. Roberts


### Book Reviews

### ESSENTIALS OF DERMATOLOGY

**J. L. Burton, BSc, MD, FRCP,**
**Churchill Livingstone – Price –**

Books on specialities tend often to confuse and even to repel by a welter of detail of interest only to the specialist concerned, but this book with its easy readability and touches of humour will be of great value and interest to every house officer, general practitioner and general physician, and can be strongly recommended.

The stated aim of this excellent book is to provide in a readable form a firm basis of facts upon the subject of Dermatology and this it certainly achieves in a clear and practical way. The subject is divided conveniently into chapters dependent first upon disorders of the anatomical and physiological properties of the skin and then upon the many and varied causes of diseases of the skin. The descriptions of diseases and their treatment are clear and concise and well related to the diagrams and photographs.

Throughout the book Dr Burton emphasises the great importance of studying and examining and of treating the patient as a whole, including his psyche, and there are therefore very useful chapters on the relationship between the skin and immunological disorders and many important systemic diseases. In a book of such general interest it would perhaps also have seemed valuable to include a short section on the specific infections – in many of which the diagnosis is made by the changes in the skin.

The presentation of the book is worthy of its contents. It is light and easily handled and well printed and by virtue of good editing is very easy to read. The anatomical drawings are very helpful, the black and white photographs excellent and the 45 colour illustrations are of superb quality and clarity.

A. T. M. Roberts

